# Dynamic early identification of hip replacement implants with high revision rates. Study based on the NJR data from UK during 2004-2012

**DOI:** 10.1371/journal.pone.0236701

**Published:** 2020-08-04

**Authors:** Alexander Begun, Alexander J. MacGregor, Dmitri Pchejetski, Elena Kulinskaya

**Affiliations:** 1 School of Computing Sciences, University of East Anglia, Norwich, United Kingdom; 2 Norwich Medical School, University of East Anglia, Norwich, United Kingdom; Universitat Witten/Herdecke, GERMANY

## Abstract

**Background:**

Hip replacement and hip resurfacing are common surgical procedures with an estimated risk of revision of 4% over 10 year period. Approximately 58% of hip replacements will last 25 years. Some implants have higher revision rates and early identification of poorly performing hip replacement implant brands and cup/head brand combinations is vital.

**Aims:**

Development of a dynamic monitoring method for the revision rates of hip implants.

**Methods:**

Data on the outcomes following the hip replacement surgery between 2004 and 2012 was obtained from the National Joint Register (NJR) in the UK. A novel dynamic algorithm based on the CUmulative SUM (CUSUM) methodology with adjustment for casemix and random frailty for an operating unit was developed and implemented to monitor the revision rates over time. The Benjamini-Hochberg FDR method was used to adjust for multiple testing of numerous hip replacement implant brands and cup/ head combinations at each time point.

**Results:**

Three poorly performing cup brands and two cup/ head brand combinations have been detected. Wright Medical UK Ltd Conserve Plus Resurfacing Cup (cup o), DePuy ASR Resurfacing Cup (cup e), and Endo Plus (UK) Limited EP-Fit Plus Polyethylene cup (cup g) showed stable multiple alarms over the period of a year or longer. An addition of a random frailty term did not change the list of underperforming components. The model with added random effect was more conservative, showing less and more delayed alarms.

**Conclusions:**

Our new algorithm is an efficient method for early detection of poorly performing components in hip replacement surgery. It can also be used for similar tasks of dynamic quality monitoring in healthcare.

## Introduction

Hip replacement and hip resurfacing are common surgical procedures for treating osteoarthritis. Total hip arthroplasty (THA) involves removing the proximal part of femur including the femoral head with the fixation of a new, smaller artificial femoral head to the femur. The surface of the acetabulum is roughened to accept a new socket component that will articulate with the new ball component. The replacement parts can be plastic (polyethylene), metal or ceramic and are used in different combinations: metal-on-plastic (a metal ball with a plastic socket is the most widely used combination), ceramic-on-plastic, ceramic-on-ceramic or more occasionally metal-on-metal (used in younger, more active patients). Approximately 58% of hip replacements will last 25 years [1]. Resurfacing the original ball and socket with placement of metallic cap over the head of femur and a metal socket in the acetabulum is another popular surgical procedure. It carries a lower risk of dislocation and allows higher level of physical activity [2, Chapter 4].

Hip replacement and hip resurfacing surgery reduces joint pain, increases patient’s mobility and improves the quality of life. It, however carries an estimated risk of revision of 4% over 10-year period [3].

All metal on metal combinations are linked with a release of metal particles, which may cause local inflammatory reactions and have other yet unknown general side effects [4]. Hip resurfacing also carries a higher complication rate, particularly in older patients and in women. The poorer mid-term performance for these types of hip replacement means they’re being used less frequently in the UK. Currently surgeons can select from more than 200 different implants and combinations of components [3]. Some implants appear to have higher revision rates than others. In the past, some total hip replacement designs had catastrophic failures resulting in a 67% five-year revision rate for one device [5].

Surgical revision is a complex and demanding procedure that is inconvenient, traumatic, and expensive. It is therefore crucial to implement methods for early identification of implants that may have high revision rates.

Continuous monitoring of the quality of the hip prosthesis components is an important objective of the NJR. The NJR implant scrutiny group considers an implant to be a Level 1 outlier when its Patient Time Incident Rate (PTIR) is twice the PTIR of the implant group [6]. In accordance with the data from NJR, 202 different cup brands were in use during the period 2004-2012 [7]. Some of these cup brands such as DePuy Resurfacing Cup and Biomet M2A-38 were reported as poorly performing and were excluded from use after 2011. An implementation of more precise methods could help to identify poorly performing components earlier, reduce the number if hip replacement revisions and patient suffering, and improve the quality of the health care. The aim of this study was to develop a dynamic monitoring method of early identification of poorly performing brands based on the quarterly revisions data. Our method is based on the CUMulative SUM (CUSUM) analysis, sequential analysis technique based on the calculation of the cumulative sums of deviations from the target behaviour over time. This method applied to revision data is sensitive to small and medium changes in hazard ratios (HRs) of revision [8]. Time-to-revision of hip prostheses varies depending on the patient characteristics, and on the type of fixation used [9]. This necessitates the use of casemix adjusted monitoring methods. The first risk adjusted CUSUM methods for time-to-failure (survival) data were introduced by Biswas and Kalbfleisch [10]. This method was picked up by the Scottish Arthroplasty Project, where, from 2010, CUSUMs are used to monitor surgeon and unit complication rates of joint replacements. This is achieved by likelihood-based scoring method with risk adjustment for age, sex, osteoarthritis (OA) and rheumatoid arthritis (RA) [11]. Standard risk-adjusted CUSUM method involves an estimation of the control limits from the learning data set providing the gold standard performance [12]. This learning data set provides the null distribution to compare the monitored data to. The control limits for the CUSUM chart are calculated based on this null distribution to guarantee a target average run length or false alarm probability under the null hypothesis of the process in-control. This choice of the learning data set is not feasible for hip replacement data due to changes over time in the components used, surgical techniques and the casemix. We determine the control data set dynamically, as a subset of the most popular implants at that time.

We use the Cox proportional hazard model, a semiparametric method of survival analysis, for casemix adjustment. Cox regression does not make any assumptions about the underlying baseline hazard function, and its coefficients provide the estimated HRs of exposure levels compared to a baseline for the risk factors of interest [8]. The Cox regression is an effective tool for detecting significant differences in hazards across exposure levels.

We use the Weibull distribution to describe the baseline hazards. This baseline distribution well describes the failure rates of hip implants [8], [13]. An additional regression model is used to adjust for casemix the shape of this Weibull hazard. This allows the relaxation of the assumption of proportionality in the Cox regression. In hip replacement, it is likely that the surgery outcomes are similar within an operating unit. We incorporate this dependence of the outcomes for the patients from the same unit through an unobserved random frailty component. This model was described previously in Begun et al. [13].

Multiple testing problems arise when several statistical tests are performed simultaneously, and each test can produce a discovery. In our case, a large number of cup components or cup and head combinations are tested for poor performance over time, resulting in multiple streams of data. In the study we use the Benjamini-Hochberg method [14] for adjusting the False Discovery Rate (FDR). This method is not as conservative as the Bonferroni correction, and allows to find poorly performing components earlier.

Here we extend our methodology, previously described in a stationary retrospective setting in Begun et al. [13] to a dynamic detection of the high revision rates. At each time point (every quarter) we apply the CUSUM-type method to the accumulated (at that point) revision rates adjusted for casemix by the Cox regression, for detection of the poorly performing components. Additionally, the random frailty component, corresponding to the operating unit, is also taken into account. The survival model and the learning dataset are updated dynamically (at each time point) providing the null distribution needed for calculation of the limits for the CUSUM charts for multiple streams [15]. Our methodology is illustrated using the 2004-2012 hip replacement data from the UK National Joint Registry.

Overall, our methodology allowed to reliably identify three poorly performing cup brands and two cup/head brand combinations prior to their identification as outliers by NJR.

## Materials and methods

### Description of the data, inclusion and exclusion criteria

The NJR data were made available after a formal request to the NJR Research Committee. The dataset is related to the data cut used in the 10th NJR Annual Report [16]. The data were anonymised in respect to patient, to surgeon and to operating unit identifying details. Approval was obtained from Computing Subcommittee of the University of East Anglia Ethics Committee, reference number CMP/1718/F/10A. The NJR dataset provides the following four groups of variables used in the time-to-failure analysis of the hip replacements to risk-adjust the CUSUM boundaries.

- Information on procedures, such as date of operation or revision, and side;
- Institution and staff involved, such as unit and consultant IDs (anonymised), and surgeon grade;
- Hip prosthesis characteristics, such as fixation type (cemented, uncemented, hybrid, resurfacing), its components (head, cup, stem, and liner brands), head size, bearing surfaces (metal (M), polyethylene (P), ceramic (C), resurfacing (R));
- Patient characteristics, such as age, sex, ASA physical status classification [17] at 5 levels from healthy (1) to near death (5), Body Mass Index (BMI), index of multiple deprivation (IMD) [18] (a higher IMD means higher proportion of people in the area classed as deprived), and death date.

Since about a half of records had missing BMI values, this factor was excluded from further consideration. ASA scores were grouped into two categories in further analysis: ASA 1-2—normal healthy patients and patients with mild systemic disease, ASA 3-5—patients with serious, non-incapacitating systemic disease, patients with life-threatening incapacitating systemic disease and patients that are near death. We assumed that the individual outcomes of the hip replacement surgery are reported quarterly from the first quarter of 2005 until the fourth quarter of 2012. The information from the baseline year 2004 is also available. Primary cleaning included elimination of the records for duplicates, second and subsequent revisions. Next, we excluded records with missing or misreported data on date, side or time to revision, IMD and surgeon grade. We also excluded patients with bilateral operations and patients younger than 50 years at operation day. Patients operated in units with number of operations per year less than 52 in the year 2004 and less than 13 operations per quarter from 01.01.2005 until 31.12.2012 (i.e. less than once per week, on average) were also excluded.

Additionally, the cases for prostheses revised within three months of implantation were censored at the time of revision to exclude failures that might be directly attributive to surgical technique or postoperative complications. We refer to the obtained data as the “full” data set.

To select the “control” data set at each quarter, all records with implanted cup and head brand combinations in the bottom 20% in popularity in year 2004 and then the bottom 20% quarterly were excluded. If some cup and head brand combinations from the top 80% in popularity in the current quarter were not presented in the previous quarter in-control data set, the missing records with these cup and head brand combinations were added to the current in-control data set.

The complete “control” data set for the period 2004-2012 includes 211,758 records for *S*_0_ = 74 different cup brand and bearing components and 158 different cup and head brand and bearing combinations. The “full” data set includes 251,933 records for *S* = 236 different cup brand and bearing components and 1178 cup and head brand and bearing combinations. Description of the “full” and the “control” data sets is given in Table 1.

**Table 1:** Description of the “control” and “full” data sets by sex.

Variable	Statistics	“Control”			“Full”		
		M	F	All	M	F	All
Sample size	Number	83238	128520	211758	99340	152593	251933
	% by sex	39.3	60.7	100	39.4	60.6	100
Revision	Number	1215	1562	2777	1505	1926	3431
	% by sex	43.8	56.2	100	43.9	56.1	100
Death before revision	Number	7573	10001	17574	8902	11854	20756
	% by sex	43.1	56.9	100	42.9	57.1	100
Different cup brands	Number	48	48	48	158	163	166
Age	Mean	69.2	71	70.3	69	71.1	70.3
	StDev	9	9.2	9.2	9.1	9.3	9.3
IMD	Mean	19.7	19.8	19.8	19.7	19.8	19.8
	StDev	9.8	9.9	9.8	9.8	9.9	9.8
HeadSize, in mm	Mean	32.2	30	30.8	32.5	30	31
	StDev	6.3	4.2	5.2	6.9	4.4	5.7
Fixation							
Cemented	Number	32921	61798	94719	38336	72892	111228
	%	39.6	48.1	44.7	38.6	47.8	44.1
Uncemented	Number	34191	44588	78779	39790	51321	91111
	%	41.1	34.7	37.2	40.1	33.6	36.2
Hybrid	Number	12512	20640	33152	15546	26083	41629
	%	15	16.1	15.7	15.6	17.1	16.5
Resurfacing	Number	3614	1494	5108	5668	2297	7965
	%	4.3	1.2	2.4	5.7	1.5	3.2
ASA 1-2	Number	68810	106842	175652	82279	126909	209188
	%	82.7	83.1	82.9	82.8	83.2	83
ASA 3-5	Number	14428	21678	36106	17061	25684	42745
	%	17.3	16.9	17.1	17.2	16.8	17
Cup/Head bearing							
Ceramic/Ceramic	Number	13566	16782	30348	15260	19030	34290
	%	16.3	13.1	14.3	15.4	12.5	13.6
Metal/Metal	Number	3548	3713	7261	4022	4291	8313
	%	4.3	2.9	3.4	4	2.8	3.3
Polyethylene/Ceramic	Number	9181	13208	22389	11165	16324	27489
	%	11	10.3	10.6	11.2	10.7	10.9
Polyethylene/Metal	Number	51385	91838	143223	59963	108443	168406
	%	61.7	71.5	67.6	60.4	71.1	66.8
Resurfacing/Metal	Number	1565	1427	2992	2786	2185	4971
	%	1.9	1.1	1.4	2.8	1.4	2
Resurfacing/Resurfacing	Number	3993	1552	5545	6144	2320	8464
	%	4.8	1.2	2.6	6.2	1.5	3.4

### Statistical analysis

Novel dynamic risk-adjusted CUSUM-based method was used for analysis of revision rates. At each quarter, we dynamically updated the control data set, estimated the unknown parameters of the time-to-revision model under the null hypothesis of no change in revision rates, computed and tested the accumulated CUSUM scores of revision rates for multiple cup components and for cup and head combinations (for simplicity, both further referred to as components) against control revision rates adjusting for casemix and for multiple testing. This method involves an approximation of the baseline hazard function for revision rate by the Weibull hazard function, and the use of Cox’s regression with frailty term for operating unit for casemix adjustment. A more flexible parametrization of the hazard function was used to relax the assumption of proportionality of hazard functions. To that purpose, an additional regression model was used to adjust by casemix the shape of the baseline Weibull hazard. The shape regression parameters are estimated by the maximum likelihood method. The model with (gamma) frailty assumes that all patients in a unit shared the same unobserved random risk. In our previous work [13] we showed that the hazards of revision and death are independent in these data and the revision rates can, therefore, be modelled on their own. It was also shown that the Weibull model fits the revision data very well. This also holds for the current data as illustrated by the comparison of empirical and fitted cumulative hazards of revision in S1 Fig.

The following covariates were used for casemix adjustment:

- Date after 2007 (indicator of the surgery date before/after 01.01.2007; baseline value is before 2007).
- Sex (baseline value is “male”).
- Age.
- Fixation (baseline “cemented”).
- Head size, in mm.
- Bearing (baseline cup/head bearing is “Ceramic/Ceramic”).

Our initial models also included ASA score, IMD and surgeon grade, however these proved not to be significant for time to revision, though patients with serious disease (ASA P3-P5) and patients from areas with high deprivation (IMD 4-5) had increased hazards of death [13]. The baseline hazard function under the null hypothesis corresponds to the hazard function of the Weibull distribution with the shape parameter *k* and the scale parameter λ, and the baseline hazard function under the alternative hypothesis is proportional to the baseline hazard times the target hazard ratio. This hazard ratio represents the minimum departure from the acceptable failure rate that we want to detect.

The risk-adjusted CUSUM method specifies the target hazard ratio of revision for the tested components in comparison to the revision rates in control data set (after adjustment for casemix). At each quarter, and for each component, non-parametric bootstrap with 800,000 replications is used to obtain in-control distribution for its CUSUM scores, and then to compare the observed score to this distribution. The Benjamini-Hochberg false discovery rate (FDR) procedure at the level *α* = 1/160 = 0.00625 was used to adjust the resulting *p*-values for multiple testing across components within the quarter. The number of components is 236 for cups and 1178 for cup/head combinations. We have set the target hazard ratio to *HR* = 1.5 for all CUSUM charts. An alarm is issued for a component if its adjusted p-value at a current quarter is below *α*. Our choice of the FDR level guarantees the in-control average run length (ARL), i.e. the expected time before a false alarm, close to 1/*α* = 160 quarters or 40 years [19]. For comparison, we also provide results for a twice larger FDR, resulting in ARL of 20 years.

The Akaike Information Criterium (AIC) and Bayesian Information Criterium (BIC) were used to compare the goodness-of-fit of the models. To assess the predictive value of our models, we also calculated the Harrell’s concordance index [20], [21] between the predicted and the observed survival. Concordance of 70% or above is considered good for survival models.

Statistical details of our approach were described in detail in Begun et al. [13] for a stationary setting. The formula for calculating of the CUSUM scores and the dynamic algorithm for detecting the poorly performing components are given in S1 and S2 Appendices. Data analysis was performed in R version 3.3.2 [22] using the University of East Anglia 140-computer-node High Performance Computing (HPC) Cluster, providing a total of 2560 CPU cores, including parallel processing and large memory resources.

## Results

### Data characteristics and dynamic time-to-revision modelling

In accordance with Table 1, about 61% of the patients in “control” and “full” data sets are females. The proportion of female patients undergoing revisions is slightly lower, at 56%. The mean age of the patients at the date of operation is 70.3 years but females are approximately 2 years older than males. Head size is about 2.5 mm greater, on average, for male patients. ‘Cemented’ is the most frequent type of fixation in both sexes with 44.7% and 44.1% in the “control” and the “full” data sets, respectively. More than 80% of the patients were healthy or had mild systemic disease (ASA 1-2) at the date of operation. Polyethylene/Metal is the most frequent type of bearing with more than 66% of cases.

**Fig 1:**
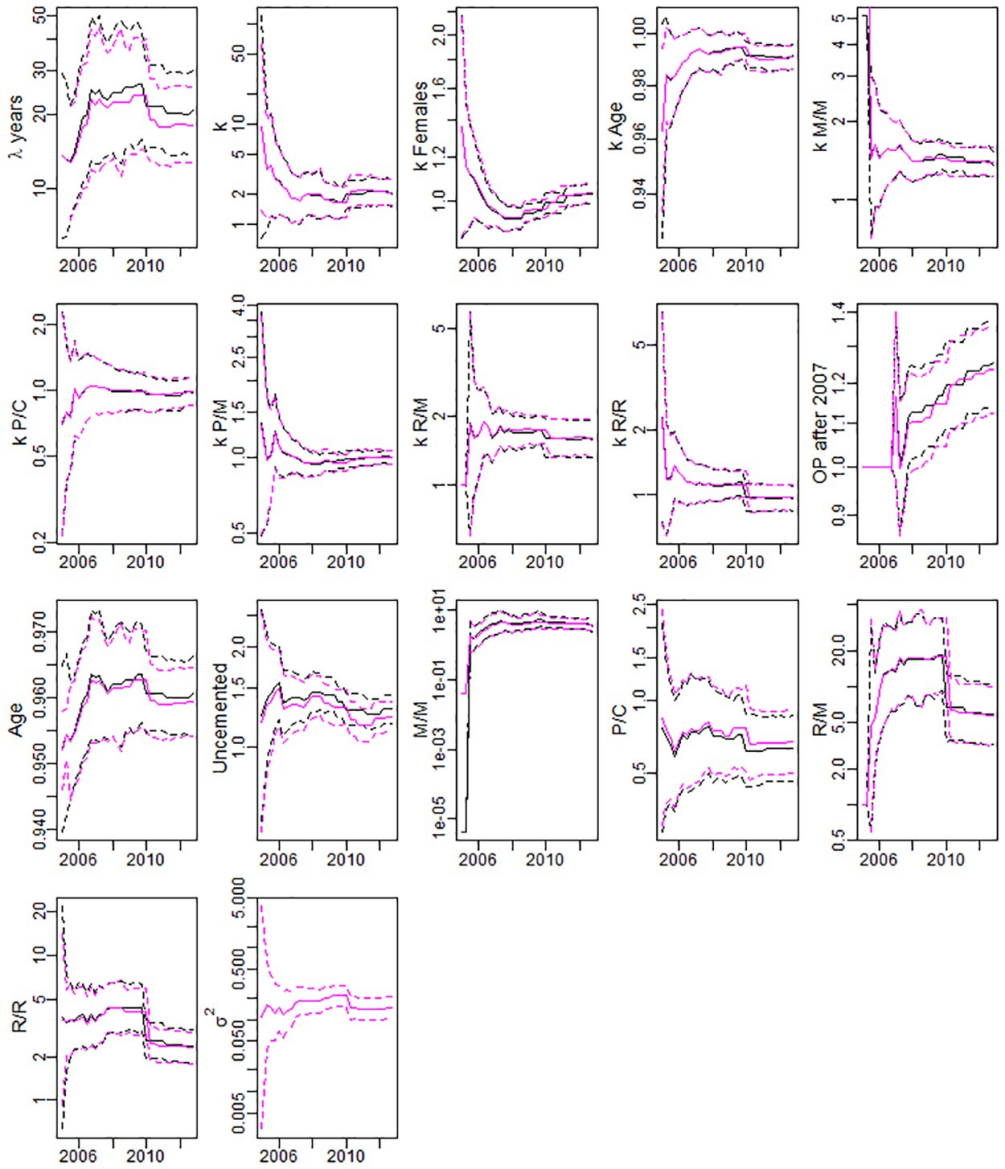
Dynamical changes in the parameter estimates of the survival models (solid) and their confidence intervals (dashed) over 2004-2012. Model with/without frailty (magenta/black). Abbreviations for cup/head bearings: C—ceramic, M—metal, P—Polyethylene, R—Resurfacing.

The casemix adjustment is performed dynamically, and we provide the evolution of the parameter estimates for the survival models with/without frailty term in Fig 1, and the parameter estimates of the respective final models in Table 2.

**Table 2:** Parameter estimates for the final survival model, at quarter 4, year 2012.

Variable	No frailty Estimate	CI	Frailty Estimate	CI
Sample size	207222		208199	
Number of non-censored	2395		2439	
**Weibull baseline hazard parameters**				
λ (years)	20.59	14.12—30.01	18.05	12.7—25.64
*k*	2.015	1.489—2.726	2.07	1.534—2.792
Frailty variance *σ*^2^			0.1444	0.1017—0.2051
**Cox’s regression parameters**				
Operation Date from 2007	1.255	1.142—1.38	1.235	1.123—1.359
Age	0.9605	0.9548—0.9662	0.9592	0.954—0.9645
Uncemented	1.302	1.178—1.439	1.231	1.104—1.373
Cup/Head Metal/Metal	3.502	2.439—5.027	3.821	2.714—5.378
Cup/Head Polyethylene/Ceramic	0.6365	0.4674—0.8668	0.6742	0.4997—0.9095
Cup/Head Resurfacing/Metal	5.933	3.244—10.85	5.664	3.198—10.03
Cup/Head Resurfacing/Resurfacing	2.367	1.825—3.069	2.309	1.801—2.962
**Shape regression parameters**				
*k* Females	1.031	0.9923—1.07	1.038	0.9968—1.08
*k* Age	0.9913	0.9868—0.9958	0.9908	0.9863—0.9953
*k* Cup/Head Metal/Metal	1.344	1.18—1.532	1.393	1.231—1.578
*k* Cup/Head Polyethylene/Ceramic	0.9743	0.8442—1.125	0.9817	0.8499—1.134
*k* Cup/Head Polyethylene/Metal	0.9961	0.9391—1.057	0.9976	0.9358—1.063
*k* Cup/Head Resurfacing/Metal	1.589	1.317—1.917	1.615	1.338—1.949
*k* Cup/Head Resurfacing/Resurfacing	0.964	0.8433—1.102	0.968	0.8465—1.107
**Goodness-of-Fit**				
LogLik	-29638.01		-30065.99	
AIC	59308.02		60165.99	
BIC	59471.88		60340.17	

From Fig 1, it can be seen that the majority of the parameter estimates stabilize by 2008-2010. There is also a clear upward trend in the contribution of post-2007 surgery data. These changes in parameters demonstrate the importance of our dynamic approach.

**Fig 2:**
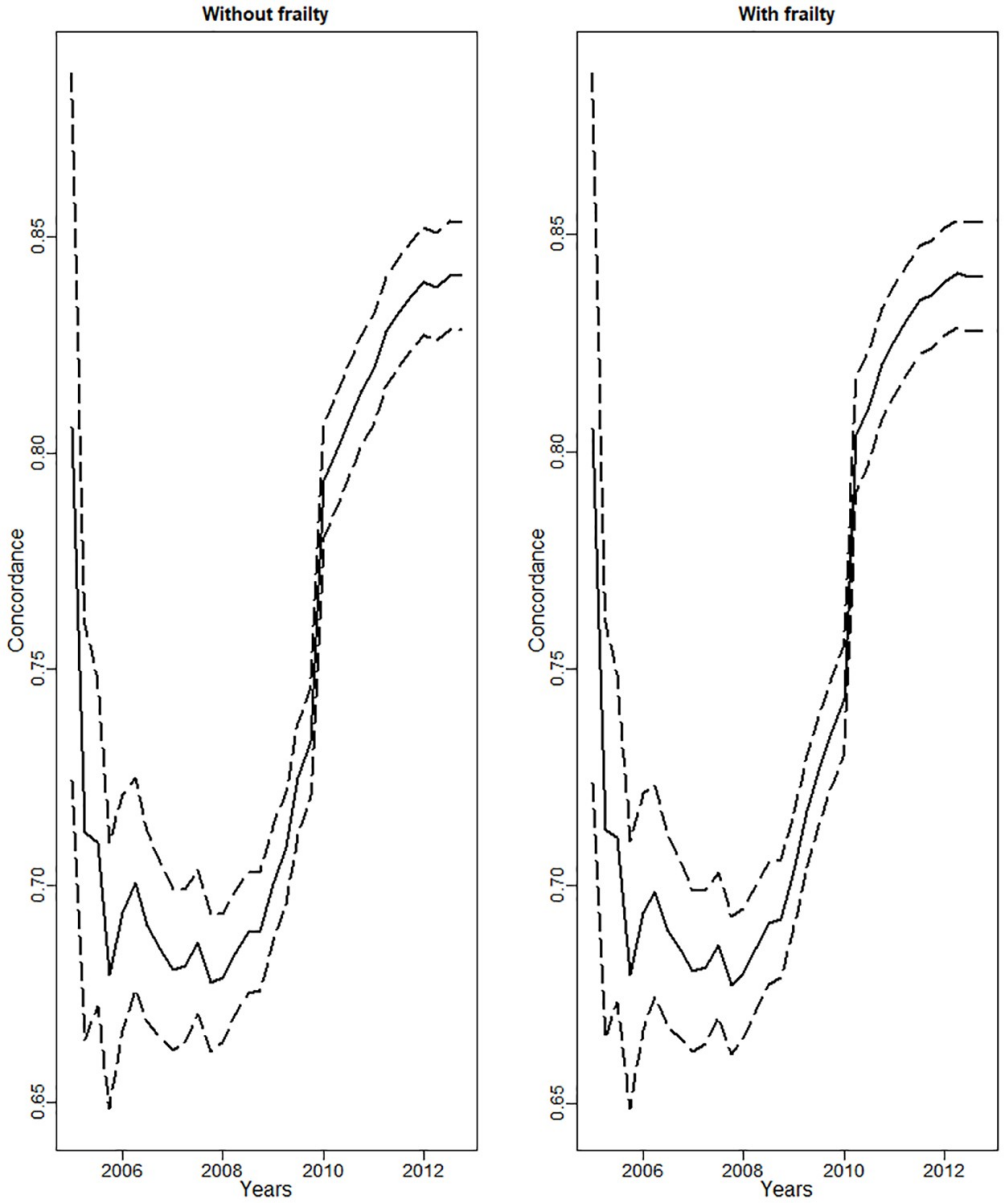
Dynamical changes in the estimated concordance of the survival models (solid) and its confidence interval (dashed) over 2005-2012.

Evolution in prognostic quality of our models as measured by concordance is given in Fig 2. It improves over time and is somewhat higher for cup components than for cup and head combinations, but in both cases it reaches 70% by 2009, and 80% by 2011 at the latest.

### Predictors of the revision

The parameter estimates of the final (for quarter 4 of 2012) time-to-revision models with/without frailty are given in Table 2. In Table 2, parameters *k* and λ are the shape and scale parameters of the baseline Weibull distribution, i.e. that for pre-2007 operations on male patients with cemented ceramic/ceramic implants. We also have found a significant random effect of units, with the estimated frailty variance *σ*^2^ equal to 0.144 with confidence interval of (0.102–0.205), i.e. the hazard of revision differed by units. The subsequent seven parameters are from the Cox’s regression. The hazard of revision significantly decreased with age. Uncemented hip prostheses had an increased hazard of revision compared to cemented fixation. Prostheses with cup/head bearings “Metal/Metal”, “Resurfacing/Metal”, and “Resurfacing/Resurfacing” had significantly increased risk of revision compared to “Ceramic/Ceramic” prostheses. Only “Polyethylene/Ceramic” bearing significantly decreased the risk of revision compared to the baseline bearing. These results agree with the findings by [6]. Those patients who underwent the surgery after 01.01.2007 had an increased hazard of revision. This may reflect the fact that early revisions were missed by the NJR due to poor data quality in the early years.

The next seven parameters in Table 2 correspond to the shape regression parameters. Out of those, *k* Age, *k* Cup/Head Metal/Metal, and *k* Cup/Head Resurfacing/Metal correspond to significant changes in the shape of the baseline hazards for the respective variables.

**Fig 3:**
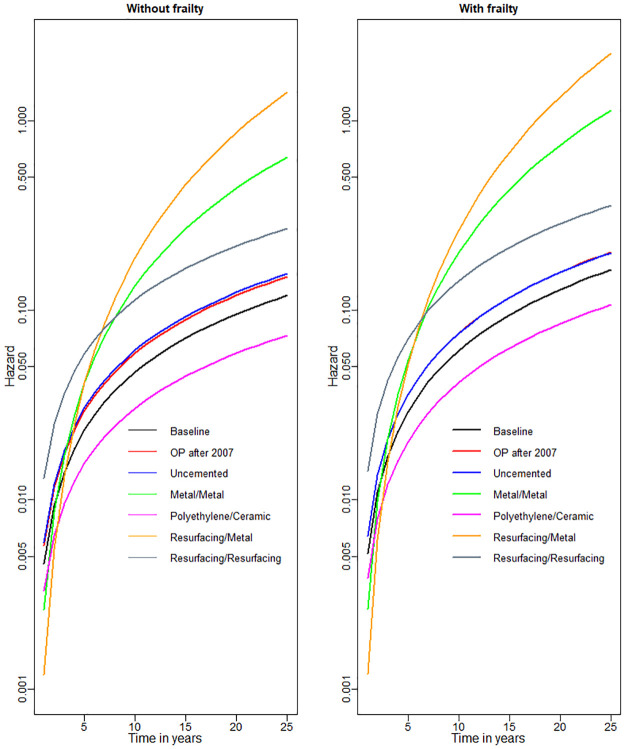
Hazard functions for different types of implant bearings. Baseline corresponds to date of operation pre 2007, male sex, cemented implant and “Ceramic&Ceramic” bearing.

Resulting estimated hazard functions for different types of implant bearings are given in Fig 3 for the models without/with frailty. The hazards cross in the first 5-10 years, demonstrating non-proportional hazards allowed by our modelling approach. For instance, the resurfacing/metal implants have the lowest revision rates in the first 3 years, but the highest rates after approximately 8 years. Metal-on-Metal prostheses demonstrate similar pattern. Polyethylene/Ceramic and, to a lesser degree, ceramic/ceramic bearings appear to be the best options overall.

### Identified out-of-control implants

**Fig 4:**
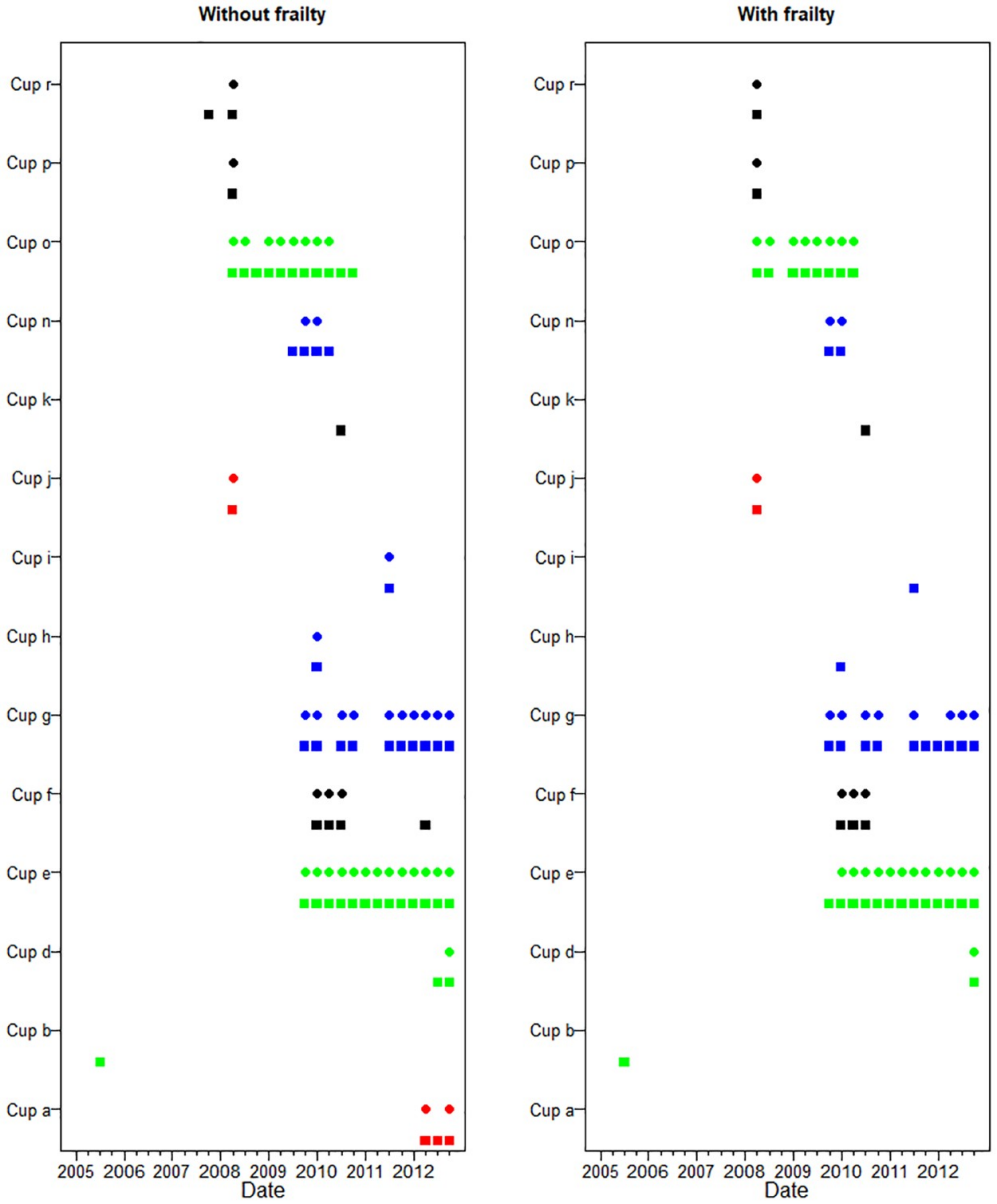
Dates of alarm by cup brands and bearing. Symbols ●/■ stand for ARL = 40/20 years. Colors ‘black’, ‘red’, ‘blue’, ‘green’ correspond to the bearings ‘Ceramic’, ‘Metal’, ‘Polyethylene’, and ‘Resurfacing’, respectively.

Next we report on the results of the CUSUM monitoring. S1 and S2 Tables provide the coding used for the names of cup and head components which generated at least one alarm. Alarms triggered by cup components during the period 2005-2012 for models without/with frailty are depicted in Fig 4. After the first alarm signal, the data for respective cup components were excluded from the control data set. The quarters when the first alarms were issued and the numbers of alarms are given in Table 3.

**Table 3:** Cup brands which triggered alarms when using CUSUM method without/with frailty during 2005-2012.

Cup brand	Bearing	ARL = 40 years		ARL = 20 years		
		Date (Y(Q))	# of alarms	Date (Y(Q))	# of alarms	# of patients
Cup a	M	2012(2)/-	2/0	2012(2)/-	3/0	774
Cup b	R		0/0	2005(3)/2005(3)	1/1	1710
Cup d	R	2012(4)/2012(4)	1/1	2012(3)/2012(4)	2/1	1299
Cup e	R	2009(4)/2010(1)	12/12	2009(4)/2009(4)	13/13	1752
Cup f	C	2010(1)/2010(1)	3/3	2010(1)/2010(1)	4/3	687
Cup g	P	2009(4)/2009(4)	10/8	2009(4)/2009(4)	10/10	2324
Cup h	P	2010(1)/-	1/0	2010(1)/2010(1)	1/1	32
Cup i	P	2011(3)/-	1/0	2011(3)/2011(3)	1/1	21
Cup j	M	2008(2)/2008(2)	1/1	2008(2)/2008(2)	1/1	59
Cup k	C		0/0	2010(3)/2010(3)	1/1	35
Cup n	P	2009(4)/2009(4)	2/2	2009(3)/2009(4)	4/2	480
Cup o	R	2008(2)/2008(2)	8/8	2008(2)/2008(2)	11/8	550
Cup p	C	2008(2)/2008(2)	1/1	2008(2)/2008(2)	1/1	360
Cup r	C	2008(2)/2008(2)	1/1	2007(4)/2008(2)	2/1	502

To illustrate the importance of dynamic modelling, four coefficients corresponding to shape and Cox regression parameters to do with resurfacing in Fig 1 noticeably drop in 2010, whereas the concordance considerably improves (Fig 2). This is due to detection of DePuy ASR Resurfacing Cup (cup e) as out-of-control in quarter 4 of 2009, and the subsequent removal of this cup (1447 patients) from in-control data-set. Overall there were 7985 patients who have undergone resurfacing by that time, so 18.1% of all patients with resurfacing were excluded. This resulted in the reduction of the estimated risk of resurfacing in the model, and the overall improvement of its quality as reflected in the surge in concordance.

In Table 3, only three cups provide stable multiple alarms over the period of a year or longer. These are Wright Medical UK Ltd Conserve Plus Resurfacing Cup (cup o), DePuy ASR Resurfacing Cup (cup e), and Endo Plus (UK) Limited EP-Fit Plus Polyethylene cup (cup g). For these three cups, the results for the analyses with/without frailty are very similar, differing at most by one quarter. Clinical characteristics of cup brands which triggered alarms in the model with frailty are provided in S3 Table.

As expected, the reduction of ARL from 40 to 20 years resulted in a larger number of alarms. Note, for instance, continuous alarms at ALR = 20 for cup o during 11 quarters from quarter 2 of 2008, accompanied by 8 alarms at ALR = 40.

There were also eight cup components which produced at most one alarm in the analysis with ARL = 40 years. Four of these eight cup components had a very small number of patients (less than 60). Seven of these alarms were a one-off signals and are likely not to be of concern. One (cup d) signalled only at the end of our study and should be subject to further monitoring. There were also three cup components with two or three alarms. These are Endo Plus (UK) Limited EP-Fit Plus ceramic cup (cup f), Waldemar Link Interplanta Polyethylene cup (cup m) and Biomet M2A 38 metal cup (cup a). The first two also produced alarms in the analysis with frailty, and the third produced two alarms in the model without frailty only at the very end of the study period, in the quarters 2 and 4 of 2012. All three would require further investigation.

We have also applied CUSUM monitoring to the cup and head brand combinations. Alarms issued for the cup/head combinations are given in S4 Table and in S2 Fig. Stable alarm signals by models with and without frailty were issued only for DePuy ASR Hip Resurfacing System and for DePuy ASR XL Acetabular System, both using the same acetabular component. However, the first alarm for the latter was triggered a year later, perhaps due to its later introduction into practice.

**Fig 5:**
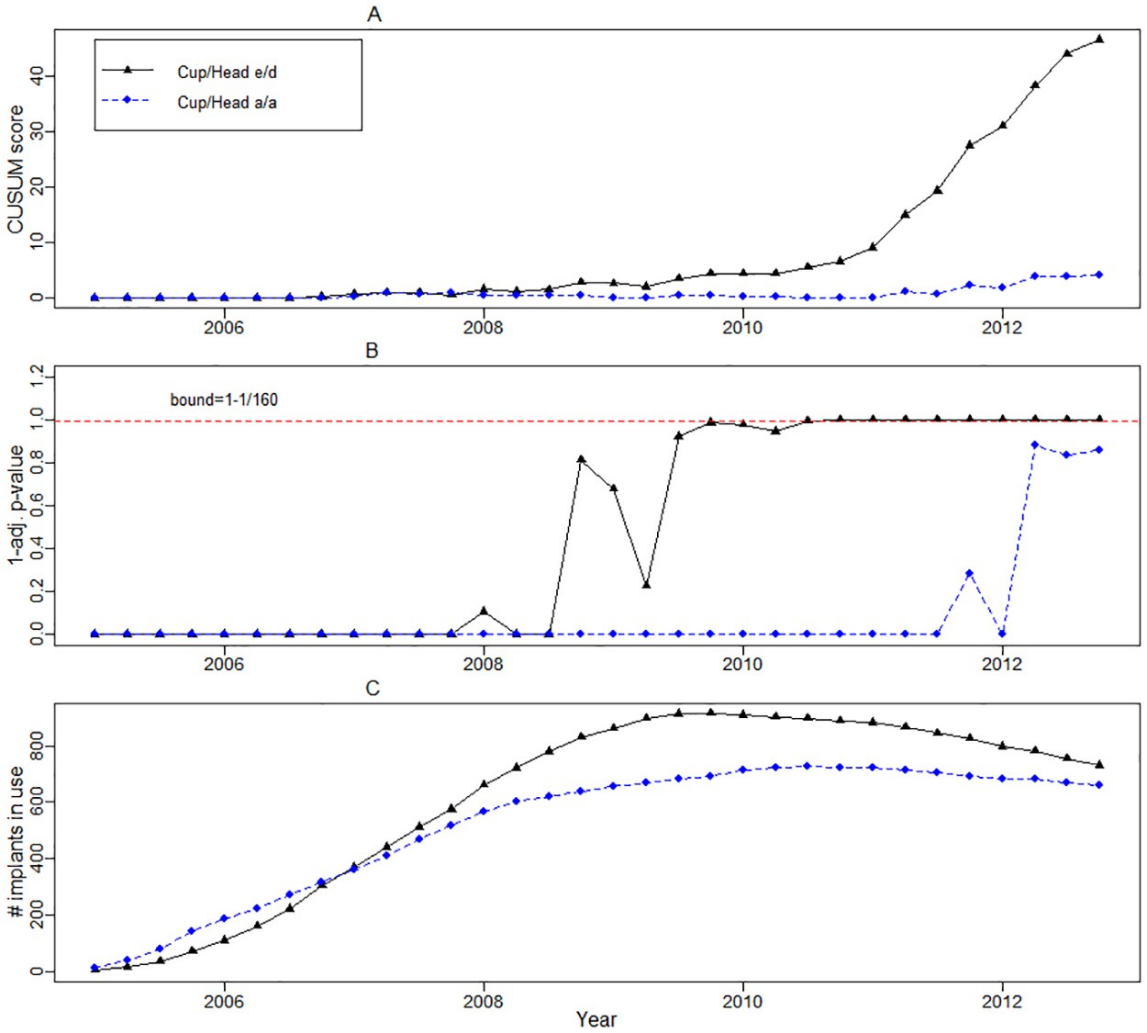
Dynamics of the CUSUM scores and adjusted p-values for two cup and head combinations. A: CUSUM scores from the model with frailty. B: 1 minus adjusted p-values for CUSUM scores, red dashed line is at the 1 − 1/160 level corresponding to ARL of 40 years. C: number of implants in use.

As an example, the CUSUM scores and the respective adjusted p-values from the model with frailty for two cup and head combinations are given in Fig 5. Recall that the alarm signals are triggered in each quarter for the components with the adjusted *p*-values below the FDR level *α* = 1/160. Combination e/d is the DePuy ASR Resurfacing System which trigerred the alarm in the 3rd quarter of 2010. Combination a/a is the Biomet M2A 38 Cup/Biomet Head which was “in-control” throughout the study in the model with frailty, but signalled at quarter 4 of 2012 in the model without frailty.

## Discussion

In hip replacement surgery, the continuous monitoring of the revision experience of hip prostheses is necessary due to delayed outcomes after the introduction of new brands into practice. CUSUM charts are a useful tool for early detection of changes in the revision rates after hip replacement because the small to medium increases in hazard ratios result in detectable cumulative effects. In the standard applications of the CUSUM-based monitoring, the learning data set required for the model identification is usually chosen from a preceding calibration period, and the obtained control parameters are used for comparison with the new data in perpetuity. This assumes the stationarity of the process being monitored. However, hip replacement practice is extremely dynamic, with changing casemix, new brands, new techniques and new prostheses appearing continuously. Therefore we developed a novel dynamic risk-adjusted CUSUM-based method for real-time analysis of the revision rates, where the new data is regularly updated. At each quarter, we dynamically updated the control data set, estimated the unknown parameters of the time-to-revision model under the null hypothesis of no change in revision rates, computed and tested the accumulated CUSUM scores of revision rates for multiple cup components and cup and head brand combinations against control revision rates adjusting for casemix and for multiple testing. We also explored an option of adjusting for unobserved covariates by adding a random shared frailty term for a unit. Ignoring the unobserved unit effects can lead to biases and underestimation of the variability [23].

In the dynamic setting, the setup phase of the monitoring process is not greatly separated from the subsequent monitoring; its main objective is to develop a dynamic definition of in-control process and a comprehensive list of covariates for the casemix-adjusting model which is refitted iteratively on the accumulating data. In this sense, the setup phase concludes when the concordance achieved by the model is sufficiently high and sufficiently stable. However, the coefficients of the model (and therefore the casemix adjustment) change dynamically, reflecting the changing casemix.

Flexible parametrization taking into account possible influence of observed covariates on the shape and the slope parameters of the revision hazard functions as well as inclusion of the random effects (frailties), accommodate non-proportional hazards and improve the fit of our models to observed data. Sex, age, fixation, bearing surfaces and the date of operation were significantly associated with the life-time of the hip prosthesis, in agreement with the findings by NJR (2017). These effects were robust against the frailty settings. This may be due to the relatively small variance of the frailty term. However, the CUSUM monitoring using the model with frailty is somewhat more conservative, i.e. the alarm signals are issued at later times.

Our method generated quite a few alarms for several cups and cup and head brand combinations. However only three cups and two cup and head brand combinations produced stable alarm signals over time. The majority of the other alarms were triggered only once or twice. A one-off signal is often due to a very small number of patients, so that the failure rate is unlikely to be stable or robust. A limitation of our approach is that it is does not take into account the fact that the choice of implant might be determined by patient characteristics. Even though each signal merits clinical review as it is vital to establish the reason for the performance assessment change at each alarm, we conjecture that only the alarms for populated components and repeated at least 2-3 times are of the real concern. Reducing the ARL may provide an extra information on the stability of the signal.

From 2009 to 2014, three hip acetabular components were reported as Level 1 outliers by NJR [24]. Our method issued an alarm for two out of these three cup components at the same time or earlier. One of these was the DePuy ASR Resurfacing Cup producing the stable alarm signal from quarter 4 of 2009. It was also a part of the two only cup and head brand combinations producing stable alarms. It was first notified by NJR in April 2010. We also found an alarm for Biomet M2A-38 metal cup by the end of our study, in quarters 2-4 of 2012. It was first identified as an outlier by the NJR only in 2014. The third cup identified by NJR, the Ultima MoM cup, had just 192 patients implanted before 2006, and wasn’t picked up by our method. However, the Wright Medical Conserve Plus resurfacing cup (another metal-on-metal cup) signalled in our study early, in quarter 2 of 2008, it was identified by [7] as having the second highest failure rate after the DePuy ASR. The Corin Cormet 2000 Resurfacing Cup shared the third highest failure rate (Tables 3.23, 3.24, [7]) and it signalled in our study in the quarter 4, 2012, whereas it was notified as an outlier by NJR only in 2015. Finally, the EP-Fit Plus cementless cup by Smith & Nephew which produced an alarm in quarter 1, 2010, was identified by NJR as having the highest failure rate among uncemented stems and cups (Tables 3.21-3.22, [7]).

We were less successful when trying to identify poorly performing cup and head brand combinations. This is due to the sheer volume of the comparisons, resulting in extremely low FDR rates for individual combinations, and smaller numbers of patients.

Overall, our method appears to be a useful addition to the real-time monitoring of the hip prostheses quality by NJR. It would be straightforward to extend it to two-sided monitoring to identify both poorly and unusually good performances. It also may be used for similar monitoring tasks in other applications.

Further development of the dynamic CUSUM-based methodology is needed to adapt our approach to real-time applications. In particular, more sophisticated methods are required to adjust for multiplicity when testing hundreds of various implant components and combinations. This was our motivation for adopting the FDR-based methodology. However, as was pointed out by a referee, step 6 in our algorithm S2 Appendix is a heuristic extension to FDR of the approach in [19] and needs to be tested by simulation and refined if required. We intend to address this and further challenges elsewhere.

## Supporting information

S1 Appendix Calculation of the CUSUM scores. (PDF)

S2 Appendix Algorithm. (PDF)

S1 Table Abbreviations for cup brands. This table contains full names and abbreviations (cups a to r) and bearing types for cup brands which triggered alarms in 2005-2012.
(PDF)

S2 Table Abbreviations for head brands. This table contains full names and abbreviations (heads a to l) and bearing types for head brands which triggered alarms in 2005-2012.
(PDF)

S3 Table Characteristics of cup brands which triggered alarms in the model with frailty at ARL = 40 years. For cup brands which triggered alarms in 2005-2012, this table compares casemix characteristics and estimated hazards at the time of the first alarm to their overall average values.
(PDF)

S4 Table Cup/head combinations which triggered alarms in 2005-2012 when using CUSUM method without/with frailty. This table provides the time of the first alarm and the number of alarms for cup/head combinations which triggered alarms in 2005-2012.
(PDF)

S1 Fig Empirical and fitted cumulative hazards of revision. This figure depicts averaged empirical and fitted cumulative hazards of revision from the final survival model, at quarter 4, year 2012, with the Weibull baseline hazards.
(PDF)

S2 Fig Dates of alarms by cup/head brands and bearing. This figure depicts the alarms for each cup/head combination chronologically, for two ALR levels (ARL = 20 years and ARL = 40 years).
(PDF)
