# Supplementary material for: Dynamic early identification of hip replacement implants with high revision rates. Study based on the NJR data from UK during 2004-2012
Source: PLoS One. 2020 Aug 4;15(8):e0236701. doi: 10.1371/journal.pone.0236701 (PMC7402470; doi:10.1371/journal.pone.0236701)
Supplement: S1 Appendix — (PDF) [file pone.0236701.s001.pdf]

S1 Appendix. Calculation of the CUSUM scores.

For s component-specific data set s, $s = 1, ..., S$, define the sequence of the one-sided CUSUM vector-scores $W^+_{i,s}$, for time points corresponding to individual observations $i = 0, 1, ...$, by the recurrent equation

$$W^+_{0,s} = 0,$$
$$W^+_{i+1,s} = \max\{0, W^+_{i,s} + X^+_{i+1,s}\},$$

with the weight $X^+_{i,s}$ assigned to the component s at time i. This weight can be calculated as the log-ratio of the likelihood for revisions for the target hazard ratio (HR) corresponding to the hypothesis H_1 and the likelihood for revisions for $HR_0 = 1$ corresponding to the hypothesis H_0.

Following [6] we will compare the number of revisions within a certain time interval (quarter) to that expected given a target revision rate HR and the total number of component-years in the interval. It means that patients contribute to a CUSUM score until revision or censoring (death or end of follow-up).

Assume that the time-to-revision is the Weibull-distributed random variable with the cumulative hazard function given by the formula $H(t) = (t/\lambda)^k$. Assume also that the individuals are observed over the consecutive time intervals T. Consider a subset $I = I(T)$ of N_I individuals observed (using particular component) over the time interval T. In this case, for a component s, the summary scores for these I individuals, X^+_I (we omit here index s which is fixed in this derivation) can be calculated as

$$X^+_I = O_I \log(HR) - (HR - 1)E_I,$$

where O_I is the observed number of revisions occurring during the interval T and E_I is the number of revisions that would be expected in the same interval under hypothesis H_0 (see [6]). The values of E_I can be computed as follows

$$E_I = \sum_{i=1}^{N_I} \lambda^{-k} \left((t_{2i} - t_{0i})^k - (t_{1i} - t_{0i})^k \right),$$

where N_I is the number of all patients using component s during the time interval T, t_{0i} is the time of initiation (implantation) for patient i , t_{1i} is the maximum of the lower bound of interval T and the time of initiation for patient i, t_{2i} is the minimum of the upper bound

of interval T, the time of revision and the time of censoring for patient i. It is easy to see that the value of $(t_{2i} - t_{1i})$ is equal to the length of time when patient i uses component s in the time interval T.

If the gamma distributed frailty component with mean 1 and the variance σ^2 (corresponding to operating unit j) is also included in the model, we can rewrite the score formula as follows

$$X_I^+ = O_I \log(HR) - \sum_{j=1}^{J} (\sigma^{-2} + O_j) \log \left(\frac{1 + \sigma^2 HR \sum_{i \in I_j(T)} \lambda^{-k}((t_{2i})^k - t_{0i})^k - (t_{1i})^k - t_{0i})^k}{1 + \sigma^2 \sum_{i \in I_j(T)} \lambda^{-k}((t_{2i})^k - t_{0i})^k - (t_{1i})^k - t_{0i})^k)} \right),$$

where O_j is a number of revisions in the unit j during period T so that $O_I = \sum_j O_j$, $I_j(T)$ is a set of individuals from unit j using component s during the period T, $I = I(T) = \bigcup I_j(T)$, and J is the number of units.

The influence of the observed factors on the scale and shape parameters of the Weibull survival distribution can be easily taken into account in the form of the Cox-like regression. In this case we assume that the cumulative hazard function is given by

$$H(t, \mathbf{u}) = \exp(\beta \mathbf{u})(t/\lambda)^{k \exp(\beta_k \mathbf{u})},$$

where $\mathbf{u}$ stands for the vector of the covariates and β, β_k are the vectors of the Cox-regression coefficients.
