# Supplementary material for: Dynamic early identification of hip replacement implants with high revision rates. Study based on the NJR data from UK during 2004-2012
Source: PLoS One. 2020 Aug 4;15(8):e0236701. doi: 10.1371/journal.pone.0236701 (PMC7402470; doi:10.1371/journal.pone.0236701)
Supplement: S2 Appendix — (PDF) [file pone.0236701.s002.pdf]

S2 Appendix. Algorithm.

Step 1 *Initialization.* Let D_c and $D = \bigcup_s D_s$, be in-control and full data sets from year 2004, with S and S_0 components, respectively, with D_s corresponding to patients with component s, $s = 1, ..., S$. Each component corresponds to a cup brand (or cup and head combination). Set $i = 0$, denote by Q the first quarter (the 4th quarter of 2004 in our data), and choose a sufficiently large positive integer N (in our analysis, $N = 8 \cdot 10^5$.) For each component s, define a vector of cumulative scores $\mathbf{C}_s = (C_{1,s}, ..., C_{N,s})$ with $C_{j,s} = 0$, $j = 1, ..., N$, $s = 1, ..., S$. Set the false discovery rate (FDR) level equal to $\alpha = 1/160 = 0.00625$ and the target hazard ratio $HR = 1.5$. This FDR level corresponds to the average run length without false alarm under null hypothesis equal to 40 years;

Step 2 *Dynamic data update.* Set $Q = Q + 1$, $i = i + 1$ and update D_c and D extending them to quarter Q as described in subsection "Description of the data". Recall that the "control" data set generally includes only the top 80% components from each quarter and the "full" data set includes all possible components;

Step 3 *Model fitting.* Calculate the maximum likelihood estimate $\hat{\xi}$ of the vector of unknown parameters $\xi = (\ln k, \ln \lambda, \ln \sigma^2, \beta, \beta_k)$ for the data set D_c. Here k and λ are the shape and the scale parameters for the Weibull hazard function, respectivelly, σ^2 is the variance of the gamma distributed frailty with mean 1, β is the vector of the Cox-regression coefficients in the model with proportional hazards, and β_k is the vector of the Cox-regression coefficients for the shape parameter;

Step 4 *Calculation of CUSUM scores and generation of their in-control distributions by nonparametric bootstrap.* Using estimated parameters $\hat{\xi}$, calculate scores $X_{i,s}^{+}$ and $W_{i,s}^{+}$ for quarter Q as described in S1 Appendix. For each component s, $s = 1, ..., S$, choose ran-

domly, from the full data set D, N data subsets (indexed by j) of the size equal to the number of records in D_s. Calculate scores $X_{i,s,j}$, $j = 1, ..., N$, and update the vector $\mathbf{C}_s$ using the equation $C_{j,s} = \max(0, C_{j,s} + X_{i,s,j})$. The N-dimensional vector $\mathbf{C}_s$ describes the empirical distribution of the CUSUM score for the component s under the null hypothesis;

Step 5 *Components testing at FDR level* α. Calculate empirical p-values α_s, $s = 1, ..., S$, using CUSUM scores $W^+_{i,s}$ and empirical null distributions given by vectors $\mathbf{C}_s$. Arrange these p-values in increasing order $\alpha^0_1 \leqslant ... \leqslant \alpha^0_S$, and find index $k(s)$ equal to the order of the p-values α_s in the sequence $\alpha^0_1, ..., \alpha^0_S$. Find maximal $k(s) = k^0$ such that $\alpha_s \leqslant k(s)\alpha/S$. Issue alarms for all components in D such that $k(s) \leqslant k^0$. Exclude respective components from the the 'control' data set D_c, update the values of S and S_0. Go to Step 6 if $S > 0$ or terminate, otherwise;

Step 6. *Preparing the in-control distributions for the next step.* Replace $\mathbf{C}_s$ with N randomly chosen values from the set $\omega = \{C_{j,s} | C_{j,s} \leqslant q_s\}$, where q_s is the $1 - k(s)\alpha/S$ quantile in the set $\mathbf{C}_s$, $s = 1, ..., S$. Go to Step 2.

Remark 1. Alternatively, in Step 5, the adjusted p-values can be calculated as $\alpha^{adj}_S = \alpha^0_S$, $\alpha^{adj}_i = \min\{\alpha^{adj}_{i+1}, \alpha^0_i S/k(i)\}$, $i = S - 1, ..., 1$. Alarms are issued for the components s with $\alpha^{adj}_{k(s)} \leqslant \alpha$.

Remark 2. At each step i, vectors $\mathbf{W}^+_{i,s}$ and $\mathbf{C}_s$ are calculated separately for cup components and for cup and head combinations. The FRD procedure is carried out separately at level α and at $\alpha_1 = 2\alpha = 2/160 = 0.0125$. All components and/or combinations for which the alarm signals at α level are excluded sequentially from the in-control data set.

Remark 3. In each quarter Q (or step i) we deal with S groups of patients whose implants

are in use. Each group of patients relates to a specific cup (cup and head combination) s. We re-estimate the parameters of the model at the end of the quarter and, based on these estimates, construct the empirical null distribution for CUSUM scores in the next quarter for each cup (cup and head combination).
