# Supplementary material for: Dynamic early identification of hip replacement implants with high revision rates. Study based on the NJR data from UK during 2004-2012
Source: PLoS One. 2020 Aug 4;15(8):e0236701. doi: 10.1371/journal.pone.0236701 (PMC7402470; doi:10.1371/journal.pone.0236701)
Supplement: S1 Table — This table contains full names and abbreviations (cups a to r) and bearing types for cup brands which triggered alarms in 2005-2012. (PDF) [file pone.0236701.s003.pdf]

S1 Table. Abbreviations for cup brands.

Cup brand	Bearing	Cup code
Biomet M2A 38	M	Cup a
Biomet Recap Magnum	R	Cup b
Centerpulse Allofit	M	Cup c
Corin Cormet 2000 Resurfacing Cup	R	Cup d
DePuy ASR Resurfacing Cup	R	Cup e
Endo Plus (UK) Limited EP-Fit Plus	C	Cup f
Endo Plus (UK) Limited EP-Fit Plus	P	Cup g
Finsbury SLF Cementless Cup	P	Cup h
Implants International RM Cementless Cup	P	Cup i
Mathys Orthopaedics Ltd seleXys PC	M	Cup j
Mathys Orthopaedics Ltd seleXys TH	C	Cup k
Stryker Trident	P	Cup l
Stryker Tritanium	P	Cup m
Waldemar Link Interplanta	P	Cup n
Wright Medical UK Ltd Conserve Plus Resurfacing Cup	R	Cup o
Wright Medical UK Ltd Procotyl	C	Cup p
Zimmer Continuum	P	Cup q
Zimmer Trilogy	C	Cup r
