# Supplementary material for: Dynamic early identification of hip replacement implants with high revision rates. Study based on the NJR data from UK during 2004-2012
Source: PLoS One. 2020 Aug 4;15(8):e0236701. doi: 10.1371/journal.pone.0236701 (PMC7402470; doi:10.1371/journal.pone.0236701)
Supplement: S2 Table — This table contains full names and abbreviations (heads a to l) and bearing types for head brands which triggered alarms in 2005-2012. (PDF) [file pone.0236701.s004.pdf]

S2 Table. Abbreviations for head brands.

Head brand	Bearing	Head code
Biomet	M	Head a
Centerpulse	M	Head b
Corin Cormet 2000 Resurfacing Head	R	Head c
DePuy	M	Head d
DePuy ASR Resurfacing Head	R	Head e
Endo Plus (UK) Limited	C	Head f
Joint Replacement Instrumentation Ltd	C	Head g
Mathys Orthopaedics Ltd	M	Head h
Waldemar Link	C	Head i
Wright Medical UK Ltd	M	Head j
Wright Medical UK Ltd Conserve	R	Head k
Zimmer	C	Head l
