# Supplementary material for: Dynamic early identification of hip replacement implants with high revision rates. Study based on the NJR data from UK during 2004-2012
Source: PLoS One. 2020 Aug 4;15(8):e0236701. doi: 10.1371/journal.pone.0236701 (PMC7402470; doi:10.1371/journal.pone.0236701)
Supplement: S3 Table — For cup brands which triggered alarms in 2005-2012, this table compares casemix characteristics and estimated hazards at the time of the first alarm to their overall average values. (PDF) [file pone.0236701.s005.pdf]

S3 Table. Characteristics of cup brands which triggered alarms in the model with frailty at ARL=40 years

For each cup brand, the table provides bearing (R - resurfacing, C - ceramic, P- Polyethylene , M -metal), year and quarter Q of the first alarm;, number of failed implants in quarter Q, to quarter Q inclusive, and in total during 2005-2012; mean age, percentage of females and percentage of uncemented implants for failed cups in quarter Q and in total; T_Q/T_0 - ratio of mean times to failure in quarter Q to total; H_Q/H_0 - ratio of cumulative hazards in quarter Q to total estimated from the Weibull model with frailty at ARL=40 years. Alarms correspond to large proportion of failures in quarter Q and, for all but cup d, to short failure times combined with low estimated hazards.

Cup	Bearing	Year	Quarter	Implants		Failed		Mean age	Females(%)	Uncemented(%)	time	cum HR
			Q	in use in Q	in Q	to Q inc	total	Q(total)	Q(total)	Q(total)	T_Q/T_0	H_Q/H_0
d	R	2012	4	1148	9	98	98	58.8(58.7)	55.6(51)	33.3(27.6)	1.71	1.87
e	R	2010	1	1642	9	72	307	60.9(59.7)	44.4(54.4)	55.6(58.6)	0.62	0.52
f	C	2010	1	539	4	15	26	60(58.4)	25(26.9)	75(88.5)	1.01	0.88
g	P	2009	4	1578	5	22	57	66.2(67.1)	80(63.2)	100(82.5)	0.84	0.80
j	M	2008	2	15	1	2	3	66(70)	100(100)	100(100)	0.26	0.09
n	P	2009	4	308	1	10	10	56(71.9)	100(60)	0(0)	0.52	0.55
o	R	2008	2	327	3	10	37	59.3(57.5)	33.3(45.9)	0(24.3)	0.72	0.56
p	C	2008	2	18	1	1	5	64(60.4)	100(40)	100(100)	0.63	0.50
r	C	2008	2	124	1	4	5	62(60.2)	0(60)	0(40)	0.76	0.65
