# Supplementary material for: Dynamic early identification of hip replacement implants with high revision rates. Study based on the NJR data from UK during 2004-2012
Source: PLoS One. 2020 Aug 4;15(8):e0236701. doi: 10.1371/journal.pone.0236701 (PMC7402470; doi:10.1371/journal.pone.0236701)
Supplement: S4 Table — This table provides the time of the first alarm and the number of alarms for cup/head combinations which triggered alarms in 2005-2012. (PDF) [file pone.0236701.s006.pdf]

S4 Table. Cup/head combinations which triggered alarms when using CUSUM method without/with frailty during 2005-2012.

		ARL=40 years		ARL=20 years		
Cup / head brand	Bearing	First signal at YQ	# of signals	First signal at YQ	# of signals	# of patients
Cup a / Head a	M/M		0/0	2012(4)/	1/0	774
Cup c / Head b	M/M	2011(4)/2011(4)	1/1	2011(4)/2011(4)	1/1	176
Cup d / Head c	R/R		0/0	2012(4)/-	1/0	980
Cup e / Head d	R/M	2009(4)/2010(3)	13/10	2009(4)/2010(3)	13/10	979
Cup e / Head e	R/R	2011(1)/2011(1)	8/8	2011(1)/2011(1)	8/8	773
Cup f / Head f	C/C	2010(1)/2010(1)	2/1	2010(1)/2010(1)	2/1	637
Cup g / Head i	P/C		0/0	2009(2)/-	1/0	11
Cup i / Head h	P/M	2011(3)/-	1/0	2011(3)/2011(3)	1/1	10
Cup j / Head h	P/C		0/0	-/2011(2)	0/1	2
Cup l / Head i	M/M		0/0	2008(2)/-	1/0	59
Cup m / Head g	P/C	2011(3)/2011(3)	1/1	2011(3)/2011(3)	1/1	2
Cup o / Head j /	R/M	2012(4)/2012(4)	1/1	2012(4)/2012(4)	1/1	65
Cup o / Head k	R/R	2009(1)/-	1/0	2008(2)/-	4/0	478
Cup q / Head j	P/M		0/0	-/2011(2)	0/1	13
Cup r / Head l /	C/C		0/0	2007(4)/-	2/0	284
