# Supplementary material for: Dynamic early identification of hip replacement implants with high revision rates. Study based on the NJR data from UK during 2004-2012
Source: PLoS One. 2020 Aug 4;15(8):e0236701. doi: 10.1371/journal.pone.0236701 (PMC7402470; doi:10.1371/journal.pone.0236701)
Supplement: S1 Fig — This figure depicts averaged empirical and fitted cumulative hazards of revision from the final survival model, at quarter 4, year 2012, with the Weibull baseline hazards. (PDF) [file pone.0236701.s007.pdf]

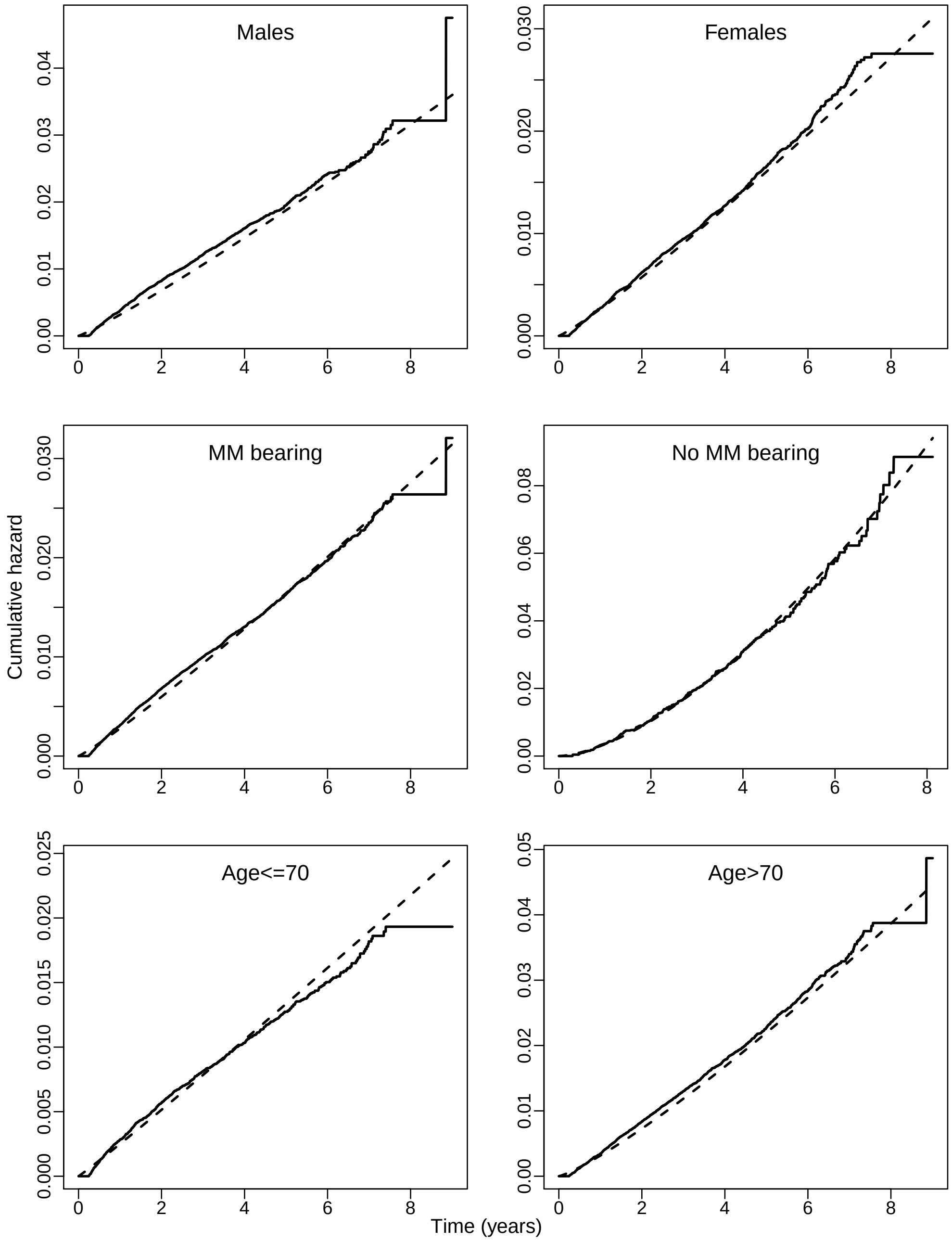

S1 Fig. Empirical and fitted cumulative hazards of revision. Averaged empirical (solid lines) and fitted (dashed lines) cumulative hazards of revision from the final survival model, at quarter 4, year 2012, with the Weibull baseline hazards.
