# Supplementary material for: Dynamic early identification of hip replacement implants with high revision rates. Study based on the NJR data from UK during 2004-2012
Source: PLoS One. 2020 Aug 4;15(8):e0236701. doi: 10.1371/journal.pone.0236701 (PMC7402470; doi:10.1371/journal.pone.0236701)
Supplement: S2 Fig — This figure depicts the alarms for each cup/head combination chronologically, for two ALR levels (ARL = 20 years and ARL = 40 years). (PDF) [file pone.0236701.s008.pdf]

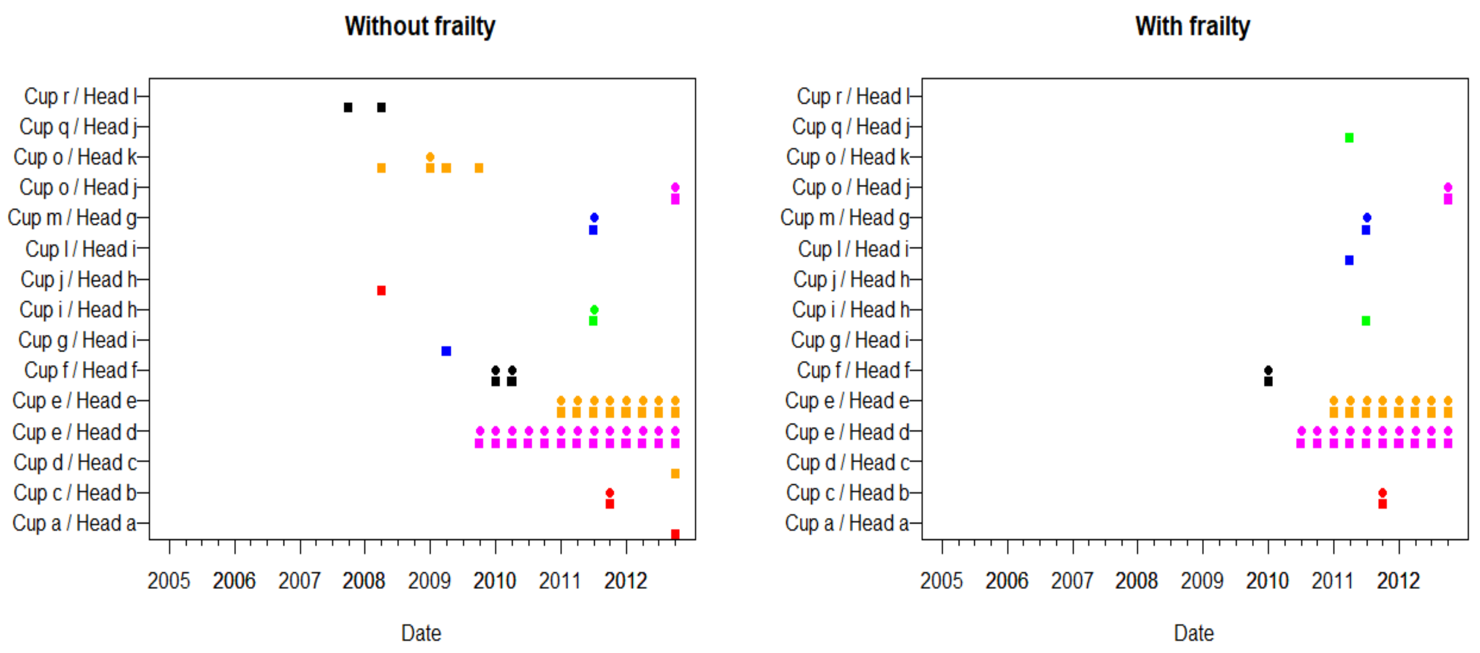

S2 Fig. Dates of alarm by cup/head brands and bearing. Symbols ●/■ stand for ARL=40/20 years. Colors 'black', 'red', 'blue', 'green','magenta' and 'orange' correspond to the bearings 'Ceramic/Ceramic', 'Metal/Metal', 'Polyethylene/Ceramic', 'Polyethylene/Metal, 'Resurfacing/Metal', and 'Resurfacing/Resurfacing', respectively.
